# Impact of an oligosaccharide-based polymer on the metabolic profiles and microbial ecology of weanling pigs experimentally infected with a pathogenic *E. coli*

**DOI:** 10.1186/s40104-023-00956-8

**Published:** 2024-01-02

**Authors:** Kwangwook Kim, Cynthia Jinno, Xunde Li, David Bravo, Eric Cox, Peng Ji, Yanhong Liu

**Affiliations:** 1 Department of Animal Science, University of California, Davis, CA 95616 USA; 2 Present Affiliation: Department of Animal Science, Michigan State University, East Lansing, MI 48824 USA; 3 Present Affiliation: Cedars-Sinai Medical Center, Los Angeles, CA 90084 USA; 4 School of Veterinary Medicine, University of California, Davis, CA 95616 USA; 5 Pancosma|ADM, 1180 Rolle, Switzerland; 6 Present Affiliation: Nutreco Exploration, Nutreco, The Netherlands; 7 Department of Virology, Parasitology and Immunology, Ghent University, 9000 Ghent, Belgium; 8 Department of Nutrition, University of California, Davis, CA 95616 USA

**Keywords:** Carbadox, Colon microbiota, Enterotoxigenic *E. coli* F18, Metabolomics, Oligosaccharide-based polymer, Weaned pigs

## Abstract

**Background:**

Our previous study has reported that supplementation of oligosaccharide-based polymer enhances gut health and disease resistance of pigs infected with enterotoxigenic *E. coli* (ETEC) F18 in a manner similar to carbadox. The objective of this study was to investigate the impacts of oligosaccharide-based polymer or antibiotic on the host metabolic profiles and colon microbiota of weaned pigs experimentally infected with ETEC F18.

**Results:**

Multivariate analysis highlighted the differences in the metabolic profiles of serum and colon digesta which were predominantly found between pigs supplemented with oligosaccharide-based polymer and antibiotic. The relative abundance of metabolic markers of immune responses and nutrient metabolisms, such as amino acids and carbohydrates, were significantly differentiated between the oligosaccharide-based polymer and antibiotic groups (*q* < 0.2 and fold change > 2.0). In addition, pigs in antibiotic had a reduced (*P* < 0.05) relative abundance of Lachnospiraceae and Lactobacillaceae, whereas had greater (*P* < 0.05) Clostridiaceae and Streptococcaceae in the colon digesta on d 11 post-inoculation (PI) compared with d 5 PI.

**Conclusions:**

The impact of oligosaccharide-based polymer on the metabolic and microbial profiles of pigs is not fully understood, and further exploration is needed. However, current research suggest that various mechanisms are involved in the enhanced disease resistance and performance in ETEC-challenged pigs by supplementing this polymer.

**Supplementary Information:**

The online version contains supplementary material available at 10.1186/s40104-023-00956-8.

## Background

The use of antimicrobials in swine production for disease prevention and growth promotion has resulted in increased public health concerns regarding antimicrobial resistance [1, 2]. Therefore, many functional substances are being investigated to enhance disease resistance in weaned pigs, while reducing the adverse environmental impacts of traditional antimicrobials [3, 4]. Dietary oligosaccharides have been shown to improve performance and health status of pigs by reducing pathogenic bacteria, such as *E. coli* and *Salmonella* [5–7], while increasing the number of generally considered beneficial bacteria in the gastrointestinal tract, such as *Lactobacillus* and *Bifidobacterium* [8, 9]. The favorable effects of dietary oligosaccharides may be attributed to several mechanisms, including serving as anti-adherence agents to prevent initial adherence and subsequent bacterial infection [10, 11], and serving as fermentable substrates to promote the growth of beneficial bacterial strains and the production of short-chain fatty acids [12, 13].

Our recent study has reported that dietary supplementation of an oligosaccharide-based polymer improved growth performance, alleviated the severity of diarrhea, and enhanced gut health in weaned pigs infected with enterotoxigenic *E. coli* (ETEC) F18 [14]. The oligosaccharide-based polymer is a customized substance containing the optimized ETEC fimbrial epitope, which is expected to disturb the binding of ETEC to their receptors in the small intestine of pigs [15, 16]. However, the exact mechanisms of action of this oligosaccharide-based polymer have not been elucidated. Carbadox is a quinoxaline-di-*N*-oxide antibiotic compound used in medicated fed for newly weaned pigs to control enteric disease. Although carbadox is not a medically important antibiotic for humans, its mutagenic property is rising public concerns [2, 17]. Therefore, the objective of this research was to investigate the impacts of this oligosaccharide-based polymer supplementation on the host metabolic profiles and colon microbiota in weaned pigs experimentally infected with ETEC F18, in comparison to carbadox.

## Materials and methods

### Animals, housing, experimental design, and diet

The protocol for this study was reviewed and approved by the Institutional Animal Care and Use Committee at the University of California, Davis (IACUC #19322; approved June 14, 2016). A total of 48 weanling pigs (Yorkshire × Landrace; initial BW: 7.23 ± 1.14 kg; 21–24 days of age) with an equal number of gilts and barrows were randomly assigned to one of four treatments in a randomized complete block design, with body weight within sex and litter as the blocks and pig as the experimental unit. There were 12 pigs per treatment group. Pigs were individually housed (pen size: 0.61 m × 1.22 m) in an environmental control unit for 18 d, including 7 d before and 11 d after the first ETEC challenge (d 0). The detailed experimental procedures were published in Kim et al. [14].

The 4 dietary treatments included: 1) Positive control: control diet; 2) Low dose oligosaccharide-based polymer (LOW): control diet supplemented with 10 mg/kg of oligosaccharide-based polymer active substance; 3) High dose oligosaccharide-based polymer (HIGH): control diet supplemented with 20 mg/kg of oligosaccharide-based polymer active substance; and 4) CAR: control diet supplemented with 50 mg/kg of carbadox. Spray-dried plasma, antibiotics, and high level of zinc oxide exceeding recommendation and normal practice were not included in the diets. The experimental diets were fed to pigs throughout the experiment and diet formulation was reported in Kim et al. [14]. The oligosaccharide-based polymer active substance was a glycoconjugate composed of blood group A6 type 1 antigen oligosaccharides grafted on a single peptide of epsilon-poly-lysine. Oligosaccharide-based polymer active substance was designed and synthesized by Elicityl (France) in cooperation with Ghent University (Dr. Eric Cox’s laboratory) and was provided by Pancosma (Geneva, Switzerland). The mean rate of conjugation is 15 moles of oligosaccharide for 1 mole of epsilon-poly-lysine. The oligosaccharide part represents 25% of the molecular weight of the oligosaccharide-based polymer.

After 7 d adaptation, all pigs were orally inoculated with 3 mL of ETEC F18 for 3 consecutive days from d 0 post-inoculation (PI). The ETEC F18 were originally isolated from a field disease outbreak by the University of Illinois Veterinary Diagnostic Lab (isolate number: U.IL-VDL # 05–27242). The ETEC F18 expressed heat-labile toxin, heat-stable toxin b, and Shiga-like toxin 2. The inoculums were prepared by the Western Institute for Food Safety and Security at the University of California, Davis, and were provided at 10^10^ CFU/3 mL dose in phosphate buffer saline. This dose caused mild diarrhea in the current study, which is consistent with our previously published research [18, 19]. Growth performance, blood profiles, and immune responses were published in Kim et al. [14].

### Sample collections

Twenty-four pigs (6 pigs per treatment) were selected randomly and euthanized on d 5 PI near the peak of ETEC infection. The remaining pigs were euthanized on d 11 PI. The selection of necropsy time was based on the results of clinical observations and immune response parameters that were reported in previously published research using the same *E. coli* strain and inoculation dose [18, 19]. Before euthanasia, pigs were anesthetized with a 1-mL mixture of 100 mg Telazol, 50 mg ketamine, and 50 mg xylazine (2:1:1) by intramuscularly injecting into the ham of the hind leg. After anesthesia, an intracardiac injection with 78 mg sodium pentobarbital (Vortech Pharmaceuticals, Ltd., Dearborn, MI, USA) per 1 kg of BW was used to euthanize each pig. Blood samples were collected from the jugular vein of all pigs without EDTA to yield serum before ETEC challenge (d 0), and on d 5, and 11 PI. Serum samples were collected by centrifuging approximately 5 mL of whole blood samples at 20 °C at 1,500 × *g* for 15 min and immediately stored at −80 °C for untargeted metabolomics analysis. Colon digesta were collected from the distal colon of pigs on d 5 and 11 PI and immediately snap-frozen in liquid nitrogen and stored at −80 °C for untargeted metabolomics and microbiome analysis.

### Untargeted metabolomics analysis

The untargeted metabolomics analysis was performed by the NIH West Coast Metabolomics Center using gas chromatography (Agilent 6890 gas chromatograph controlled using Leco ChromaTOF software version 2.32, Agilent, Santa Clara, CA, USA) coupled with time-of-flight mass spectrometry (GC/TOF–MS) (Leco Pegasus IV time-of-flight mass spectrometer controlled using Leco ChromaTOF software version 2.32, Leco, Joseph, MI, USA). Metabolite extraction was performed following procedures described previously by Fiehn et al. [20]. Briefly, frozen serum and colon digesta samples (approximately 30 μL and 10 mg, respectively) were homogenized using a Retsch ball mill (Retsch, Newtown, PA, USA) for 30 s at 25 times/s. After homogenization, a prechilled (−20 °C) extraction solution (isopropanol/acetonitrile/water at the volume ratio 3:3:2, degassed with liquid nitrogen) was added at a volume of 1 mL/20 mg of sample. Samples were then vortexed and shaken for metabolite extraction. After centrifugation at 12,800 × *g* for 2 min, the supernatant was collected and divided into two equal aliquots and concentrated at room temperature for 4 h in a cold-trap vacuum concentrator (Labconco Centrivap, Kansas City, MO, USA). To separate complex lipids and waxes, the residue was re-suspended in 500 µL of 50% aqueous acetonitrile and centrifuged at 12,800 × *g* for 2 min. The resultant supernatant was collected and concentrated in the vacuum concentrator. Dried sample extracts were derivatized and mixed with internal retention index markers (fatty acid methyl esters with the chain length of C8 to C30). The samples were injected for GC/TOF analysis, and all samples were analyzed in a single batch. Data acquisition by mass spectrometry and mass calibration using FC43 (perfluorotributylamine) before starting analysis sequences. Metabolite identifications were performed based on the two parameters: 1) Retention index window ± 2,000 U (around ± 2 s retention time deviation), and 2) Mass spectral similarity plus additional confidence criteria as detailed below (Data analysis). A detailed methodology for data acquisition and metabolite identification is described in a previously published article by Fiehn et al. [20].

### Gut microbiota in distal colon

Bacterial DNA was extracted from digesta samples using the Quick-DNA Fecal/Soil Microbe Kit (Zymo Research, Irvine, CA, USA), following the manufacturer’s instructions. Extracted bacterial DNA was amplified with PCR, targeting the V4 region of the 16S rRNA gene with primers 515 F (5′-XXXXXXXX*GT*GTGCCAGCMGCCGCGGTAA-3′) with an 8 bp barcode (X) and Illumina adapter (*GT*) and 806 R (5′-GGACTACHVGGGTWTCTAAT-3′) [21]. Amplification included thermocycling conditions of 94 °C for 3 min for denaturation, 35 cycles of 94 °C for 45 s, 50 °C for 1 min, 72 °C for 1.5 min, and 72 °C for 10 min (final elongation). To reduce PCR bias, each sample was amplified in triplicate. Each PCR reaction included 2 μL of template DNA, 0.5 μL (10 μmol/L) of barcoded forward primer, 0.5 μL (10 μmol/L) of reverse primer, 12.5 μL of GoTaq 2× Green Master Mix (Promega, Madison, WI, USA), and 9.5 μL of nuclease-free water. The triplicate PCR products were pooled and subjectively quantified based on the brightness of the bands on a 2% agarose gel with SYBR safe (Invitrogen Co., Carlsbad, CA, USA). All amplicons were then pooled at equal amounts and further purified using the QIAquick PCR Purification Kit (Qiagen, Hilden, Germany). The purified library was submitted to the UC Davis Genome Center DNA Technologies Core for 250 bp paired-end sequencing on the Illumina MiSeq platform (Illumina, Inc., San Diego, CA, USA).

The software sabre (https://github.com/najoshi/sabre) was used to demultiplex and remove barcodes from raw sequences. Sequences were then imported into Quantitative Insights Into Microbial Ecology 2 (QIIME2; version 2018.6) for downstream filtering and bioinformatics analysis [22, 23]. Plugin q2-dada2 [24] was used for quality control and constructing features. Taxonomic classification was assigned using the feature-classifier plugin trained with the SILVA rRNA database 99% Operational Taxonomic Units (OTU), version 132 [25, 26].

### Data analysis

The metabolomics data were analyzed using different modules of a web-based platform, MetaboAnalyst 5.0 (https://www.metaboanalyst.ca) [27]. Data were filtered for peaks with detection rates less than 30% of missing abundances and normalized using logarithmic transformation and auto-scaling. Mass univariate analysis was performed using one-way ANOVA followed by Tukey’s post hoc test (adjusted *P* ≤ 0.05). Fold change analysis and *t*-tests were also conducted to determine the fold change and significance of each identified metabolite. Statistical significance was declared at a false discovery rate (FDR, Benjamini and Hochberg correction; *q*) *q* < 0.2 and fold change > 2.0. Partial least squares discriminant analysis (PLS-DA) was carried out to further identify discriminative variables (metabolites) among the treatment groups. Pathway analysis and metabolite set enrichment analysis were performed on identified metabolites that had a variable importance in projection (VIP) score > 1.

Data visualization and statistical analysis for colon microbiota were conducted using R (version 3.6.1). Two alpha diversity indices, Chao1 and Shannon, were calculated using the phyloseq package. Relative abundance was calculated using the phyloseq package and visualized using the ggplot2 package in R. Relative abundance data were aggregated at various taxonomical levels. Shapiro–Wilk normality test and Bartlett test were used to verify normality and constant variance, respectively, in alpha diversity and relative abundance. Shannon index was analyzed using ANOVA with the statistical model, including sample collection days within treatment as fixed effects. Significance in Chao1 index and relative abundance was observed using Kruskal–Wallis rank-sum test followed by a Conover test for multiple pairwise comparisons using the agricolae package. Beta diversity was calculated based on the Bray–Curtis dissimilarity for principal coordinates analysis (PCoA). The homogeneity of multivariate dispersions was tested by the vegan package using the betadisper function before the adonis function was used to calculate PERMANOVA with 999 replicate permutations. Statistical significance and tendency were considered at *P* < 0.05 and 0.05 ≤ *P* < 0.10, respectively.

## Results

### Metabolite profiles in serum

**Fig. 1:**
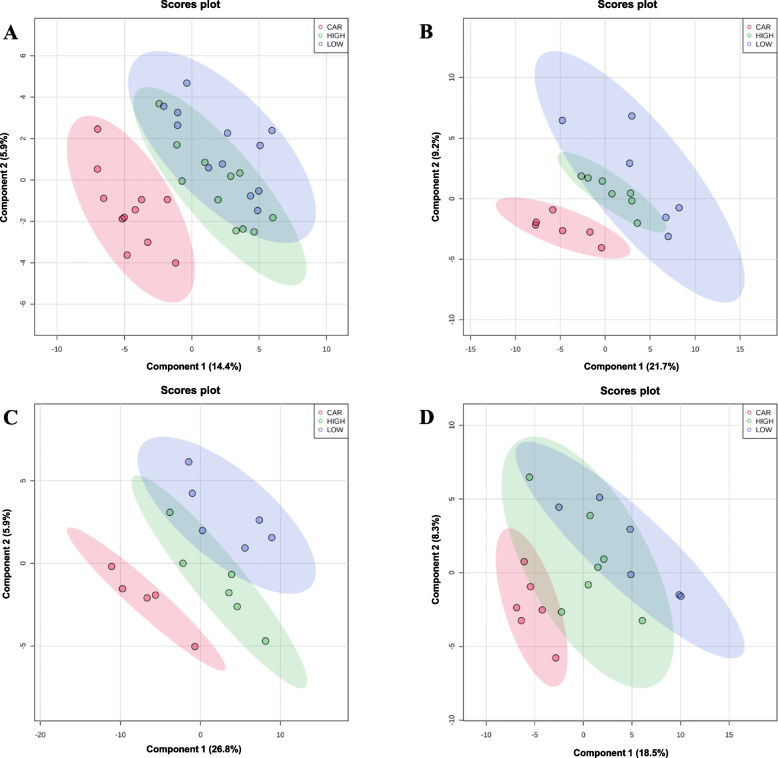
Partial least squares discriminant analysis (PLS-DA) 2D score plot of the metabolites in serum (**A** and **B**) or distal colon digesta (**C** and **D**) showed separated clusters between the 10 or 20 mg/kg oligosaccharide-based polymer active substance and CAR groups on d 5 (**A** and **C**) or d 11 (**B** and **D**) post-inoculation, respectively. LOW = Control diet supplemented with 10 mg/kg of oligosaccharide-based polymer. HIGH = Control diet supplemented with 20 mg/kg of oligosaccharide-based polymer. CAR = Control diet supplemented with 50 mg/kg carbadox. Shaded areas in different colors represent in 95% confidence interval

A total of 355 (134 identified and 221 unidentified) metabolites were detected in serum samples. Based on the statistical threshold and VIP score, inosine was up-regulated while glycerol, galactonic acid, and propylene glycol were down-regulated by CAR, compared with the pigs in CON on d 5 PI (Table 1). Supplementation of CAR changed the abundance of 3 metabolites (inosine and guanosine were up-regulated and glycerol was down-regulated) compared with LOW, while CAR changed 5 metabolites (*p*-tolyl glucuronide and inosine were up-regulated and glycerol, palmitoleic acid, and propylene glycol were down-regulated) in comparison with HIGH on d 5 PI. On d 11 PI, glycerol and inositol-4-monophosphate were enriched in the CAR group compared with CON, while chenodeoxycholic acid was reduced in the CAR when comparing with CON or HIGH, respectively. No differential metabolites were identified on d 0 before ETEC challenge among treatments. Based on the identified metabolites, a PLS-DA score plot with 95% confidence ranges (shaded areas) showed a clear separation of CAR group from LOW or HIGH at both time points (Fig. 1A and B). The PLS-DA score plots in a pairwise manner also clearly separated CON from CAR on d 5 and 11 PI (Fig. S1A and B).

**Table 1:** Serum metabolites that differed among dietary treatments

Metabolite	Fold change^1^	FDR^2^	VIP^3^
CON^4^ vs. CAR^5^, d 5 post-inoculation			
Inosine	0.41	0.089	1.67
Glycerol	2.09	0.056	1.83
Galactonic acid	2.26	0.087	1.69
Propylene glycol	2.51	0.139	1.50
LOW^6^ vs. CAR, d 5 post-inoculation			
Inosine	0.33	0.057	2.02
Guanosine	0.49	0.062	1.88
Glycerol	3.39	0.103	1.69
HIGH^7^ vs. CAR, d 5 post-inoculation			
*p*-Tolyl glucuronide	0.47	0.106	1.73
Inosine	0.47	0.132	1.65
Glycerol	2.28	0.050	2.07
Palmitoleic acid	2.45	0.179	1.50
Propylene glycol	2.82	0.106	1.75
CON vs. CAR, d 11 post-inoculation			
Glycerol	0.33	0.146	1.76
Inositol-4-monophosphate	0.48	0.168	1.67
Chenodeoxycholic acid	3.01	0.117	1.90
HIGH vs. CAR, d 11 post-inoculation			
Chenodeoxycholic acid	0.39	0.197	2.11

^1^Fold change values less than 1 indicate that the differential metabolites were reduced in the CON compared with CAR; LOW compared with CAR; or HIGH compared with CAR, respectively

^2^FDR = False discovery rate

^3^VIP = Variable importance in the projection

^4^CON = Basal nursery diet (control)

^5^CAR = Control diet supplemented with 50 mg/kg of carbadox

^6^LOW = Control diet supplemented with 10 mg/kg of oligosaccharide-based polymer

^7^HIGH = Control diet supplemented with 20 mg/kg of oligosaccharide-based polymer

**Fig. 2:**
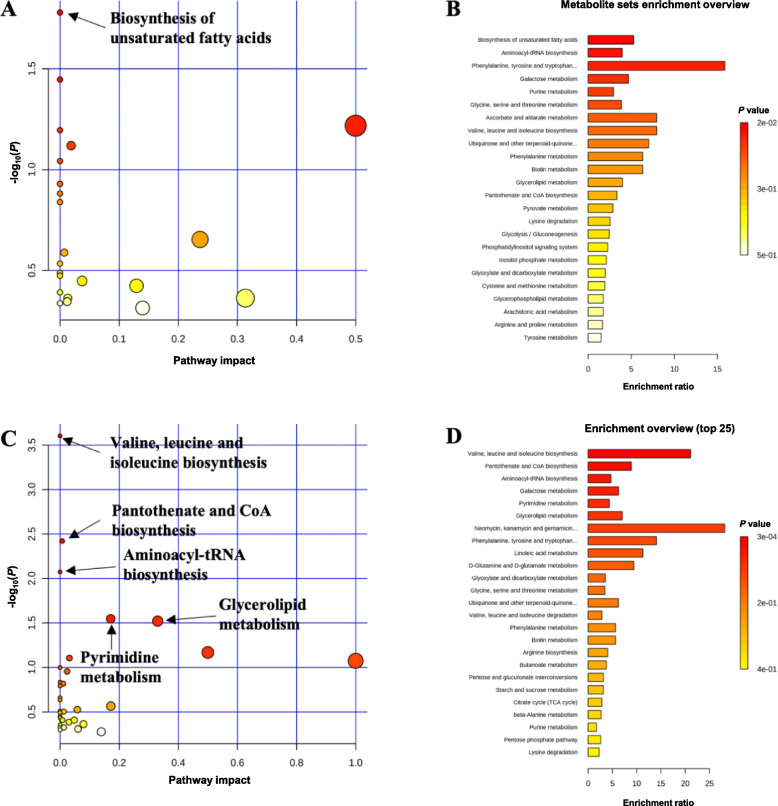
Significantly changed pathways (−log_10_(*P*) value > 1.5) in serum between oligosaccharide-based polymer groups and 50 mg/kg of carbadox on d 5 (**A**) or d 11 (**C**) post-inoculation. The *x*-axis represents the pathway impact values and the *y*-axis represents the −log_10_(*P*) values from the pathway enrichment analysis. Metabolite set enrichment analysis (**B** and **D**) shows the metabolic pathways were altered in 10 or 20 mg/kg of oligosaccharide-based polymer active substance groups compared to carbadox on d 5 or d 11 post-inoculation, respectively. Both pathway analysis and metabolite set enrichment analysis were performed using identified metabolites with VIP > 1

Pathway analysis and metabolite set enrichment analysis were performed on metabolites in serum with VIP > 1. On d 5 PI, biosynthesis of unsaturated fatty acids was the most affected metabolic pathway when comparing 10 or 20 mg/kg of oligosaccharide-based polymer with CAR (Fig. 2A and B). Aminoacyl-tRNA biosynthesis, biosynthesis of unsaturated fatty acids, phenylalanine, tyrosine and tryptophan biosynthesis, and ascorbate and aldarate metabolism were the most affected metabolic pathways when CON was compared with CAR (Fig. S2A and B). On d 11 PI, valine, leucine and isoleucine biosynthesis, pantothenate and CoA biosynthesis, aminoacyl-tRNA biosynthesis, glycerolipid metabolism, and pyrimidine metabolism were the most affected metabolic pathways when comparing 10 or 20 mg/kg of oligosaccharide-based polymer with CAR (Fig. 2C and D). Arginine biosynthesis, alanine, aspartate and glutamate metabolism, D-Glutamine and D-glutamate metabolism, nitrogen metabolism, pantothenate and CoA biosynthesis, citrate cycle (TCA cycle), and pyrimidine metabolism were the most affected metabolic pathways when CON group was compared with the CAR group (Fig. S2C and D).

### Metabolite profiles in distal colon digesta

A total of 398 (178 identified and 220 unidentified) metabolites were detected in distal colon digesta samples. On d 5 PI, tocopherol acetate was up-regulated, while arabitol, octadecylglycerol, 2-hydroxyglutaric acid, and 2-hydroxyvaleric acid were down-regulated in pigs supplemented with CAR compared with pigs in LOW (Table 2). Supplementation of HIGH enriched 11 metabolites, but reduced phosphate in colon digesta compared with CAR on d 5 PI. On d 11 PI, LOW changed 17 metabolites (10 up-regulated and 7 down-regulated) in colon digesta compared with CON, while phytanic acid was reduced in colon digesta of pigs fed with HIGH vs. LOW. Pigs in CAR had lower 6-deoxyglucose, adenosine, and fructose in colon digesta than pigs in the LOW group on d 11 PI. Nine metabolites (1 up-regulated and 8 down-regulated) were changed in the colon digesta of HIGH pigs compared with CAR on d 11 PI. No differential metabolites were identified in colon digesta when comparing CON vs. HIGH or CON vs. CAR on both time points. Based on the identified metabolites, a PLS-DA score plot with 95% confidence ranges (shaded areas) showed a clear separation between the LOW or HIGH and CAR groups at both time points (Fig. 1C and D). The PLS-DA score plots in a pairwise manner also clearly separated CON from LOW or HIGH on 11 PI (Fig. S1C).

**Table 2:** Distal colon digesta metabolites that differed among dietary treatments

Metabolite	Fold change^1^	FDR^2^	VIP^3^
LOW^4^ vs. CAR^5^, d 5 post-inoculation			
Tocopherol acetate	0.47	0.186	1.92
Arabitol	2.03	0.186	1.99
Octadecylglycerol	3.32	0.186	1.97
2-Hydroxyglutaric acid	3.62	0.186	2.09
2-Hydroxyvaleric acid	11.32	0.186	1.93
HIGH^6^ vs. CAR, d 5 post-inoculation			
Phosphate	0.32	0.164	1.60
Arabitol	1.99	1.590	1.64
Conduritol-beta-epoxide	2.02	0.164	1.56
Ferulic acid	2.19	0.140	1.80
Cellobiose	2.28	0.188	1.52
Proline	2.36	0.164	1.58
Docosahexaenoic acid	2.43	0.140	1.77
Cysteine	2.97	0.140	1.74
Octadecylglycerol	3.31	1.729	1.56
Maltotriose	4.85	0.156	1.66
Indole-3-acetate	7.97	0.164	1.57
Tranexamic acid	12.06	0.140	1.91
CON^7^ vs. LOW, d 11 post-inoculation			
Tranexamic acid	0.16	0.159	1.73
Hydroquinone	0.19	0.197	1.55
Urea	0.31	0.190	1.61
2-Hydroxyhexanoic acid	0.35	0.190	1.60
3,6-Anhydro-D-glucose	0.39	0.105	1.96
Adenosine	0.40	0.138	1.82
3,6-Anhydro-D-galactose	0.44	0.159	1.76
Fructose	0.44	0.182	1.66
Glucose	0.47	0.167	1.70
Myo-inositol	0.48	0.161	1.72
Pimelic acid	2.01	0.159	1.76
Nonadecanoic acid	2.37	0.138	1.84
Heptadecanoic acid	2.57	0.105	1.92
Pentadecanoic acid	2.58	0.105	1.94
Isopentadecanoic acid	2.66	0.052	2.15
Isoheptadecanoic acid	3.33	0.159	1.74
Uric acid	3.35	0.122	1.88
LOW vs. HIGH, d 11 post-inoculation			
Phytanic acid	0.50	0.147	2.42
LOW vs. CAR, d 11 post-inoculation			
6-Deoxyglucose	2.09	0.185	1.79
Adenosine	3.20	0.185	1.76
Fructose	4.26	0.065	1.98
HIGH vs. CAR, d 11 post-inoculation			
Fructose-6-phosphate	0.21	0.168	1.75
Xanthosine	2.02	0.170	1.67
Aminomalonate	2.03	0.168	1.74
Pyruvic acid	2.14	0.105	1.89
6-Deoxyglucose	2.31	0.170	1.66
Azelaic acid	2.50	0.053	2.02
3-Hydroxybutyric acid	2.62	0.170	1.68
Arabitol	3.12	0.168	1.76
2-Hydroxyhexanoic acid	9.76	0.170	0.89

^1^Fold change values less than 1 indicate that the differential metabolites were reduced in the LOW compared with CAR; HIGH compared with CAR; CON compared with LOW; or LOW compared with HIGH, respectively

^2^FDR = False discovery rate

^3^VIP = Variable importance in the projection

^4^LOW = Control diet supplemented with 10 mg/kg of oligosaccharide-based polymer

^5^CAR = Control diet supplemented with 50 mg/kg of carbadox

^6^HIGH = Control diet supplemented with 20 mg/kg of oligosaccharide-based polymer

^7^CON = Basal nursery diet (control)

**Fig. 3:**
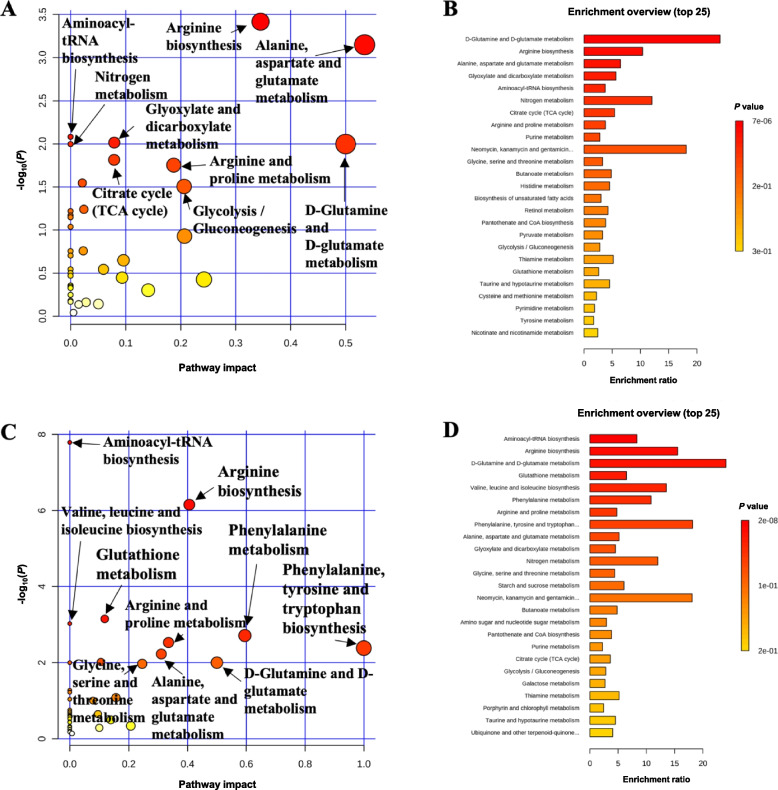
Significantly changed pathways (−log_10_(*P*) value > 1.5) in distal colon digesta oligosaccharide-based polymer groups and 50 mg/kg of carbadox on d 5 (**A**) or d 11 (**C**) post-inoculation. The *x*-axis represents the pathway impact values and the *y*-axis represents the −log_10_(*P*) values from the pathway enrichment analysis. Metabolite set enrichment analysis (**B** and **D**) shows the metabolic pathways were altered in 10 or 20 mg/kg oligosaccharide-based polymer active substance groups compared to carbadox on d 5 or d 11 post-inoculation, respectively. Both pathway analysis and metabolite set enrichment analysis were performed using identified metabolites with VIP > 1

Pathway analysis and metabolite set enrichment analysis were performed on metabolites in colon digesta with VIP > 1. On d 5 PI, arginine biosynthesis, alanine, aspartate and glutamate metabolism, aminoacyl-tRNA biosynthesis, nitrogen metabolism, glyoxylate and dicarboxylate metabolism, arginine and proline metabolism, D-glutamine and D-glutamate metabolism, citrate cycle (TCA cycle), and glycolysis/gluconeogenesis were the most affected metabolic pathway when LOW or HIGH comparing with CAR (Fig. 3A and B). On d 11 PI, aminoacyl-tRNA biosynthesis, arginine biosynthesis, valine, leucine and isoleucine biosynthesis, glutathione metabolism, arginine and proline metabolism, phenylalanine metabolism, phenylalanine, tyrosine and tryptophan biosynthesis, glycine, serine and threonine metabolism, alanine, aspartate and glutamate metabolism, and D-glutamine and D-glutamate metabolism were the most affected metabolic pathways when LOW or HIGH group was compared with the CAR group (Fig. 3C and D).

### Microbial profiles in distal colon digesta

**Fig. 4:**
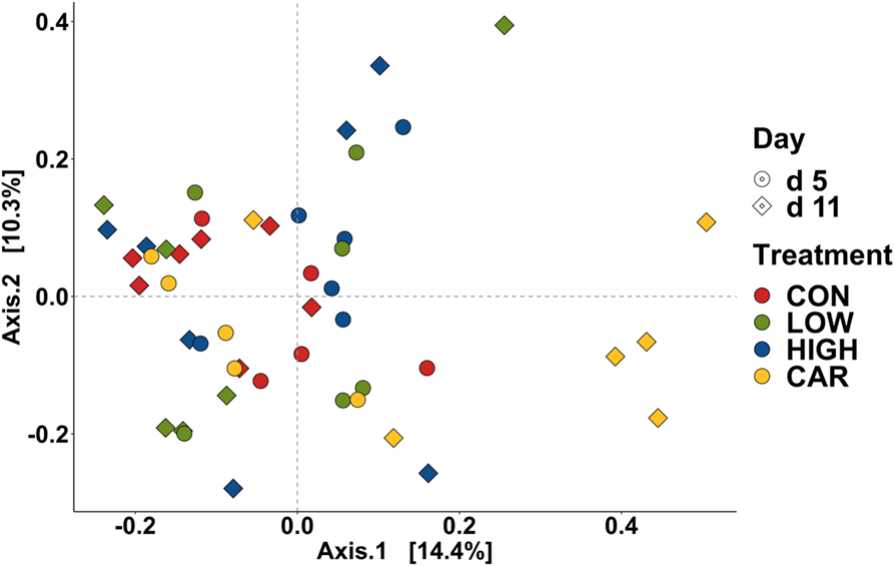
Beta diversity of the microbiota in the distal colon digesta of enterotoxigenic *E. coli* F18 challenged pigs fed diets supplemented with different dose of oligosaccharide-based polymer active substance or carbadox on d 5 and 11 post-inoculation. Data were analyzed by principal coordinate analysis (PCoA) based on the Bray–Curtis dissimilarity. Symbols indicate dietary treatments and colors indicate different sampling dates. CON = Basal nursery diet (control); LOW = Control diet supplemented with 10 mg/kg of oligosaccharide-based polymer; HIGH = Control diet supplemented with 20 mg/kg of oligosaccharide-based polymer; CAR = Control diet supplemented with 50 mg/kg of carbadox

**Fig. 5:**
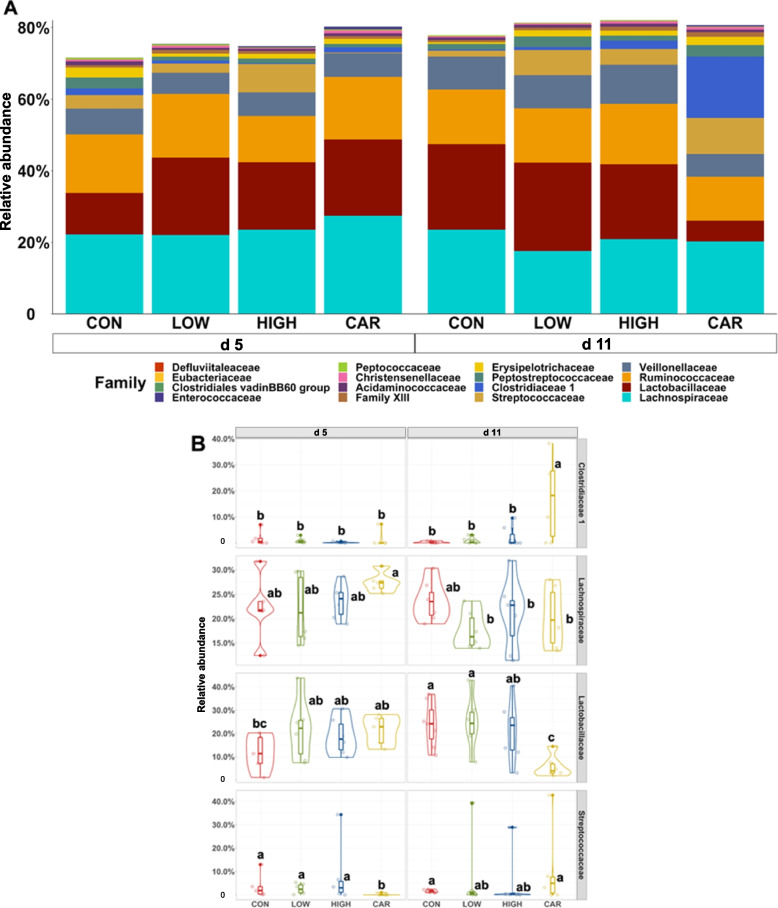
Stacked bar plot showing the relative abundance of Firmicutes family in colon digesta of enterotoxigenic *E. coli* F18 challenged pigs fed diets supplemented with different dose of oligosaccharide-based polymer active substance or carbadox on d 5 and 11 post-inoculation (**A**). Violin plot showing the relative abundance of individual bacterial phylum (**B**). ^a−c^Means without a common superscript are different (*P* < 0.05). Each least squares mean represents 6 observations. CON = Basal nursery diet (control); LOW = Control diet supplemented with 10 mg/kg of oligosaccharide-based polymer; HIGH = Control diet supplemented with 20 mg/kg of oligosaccharide-based polymer; CAR = Control diet supplemented with 50 mg/kg of carbadox

**Fig. 6:**
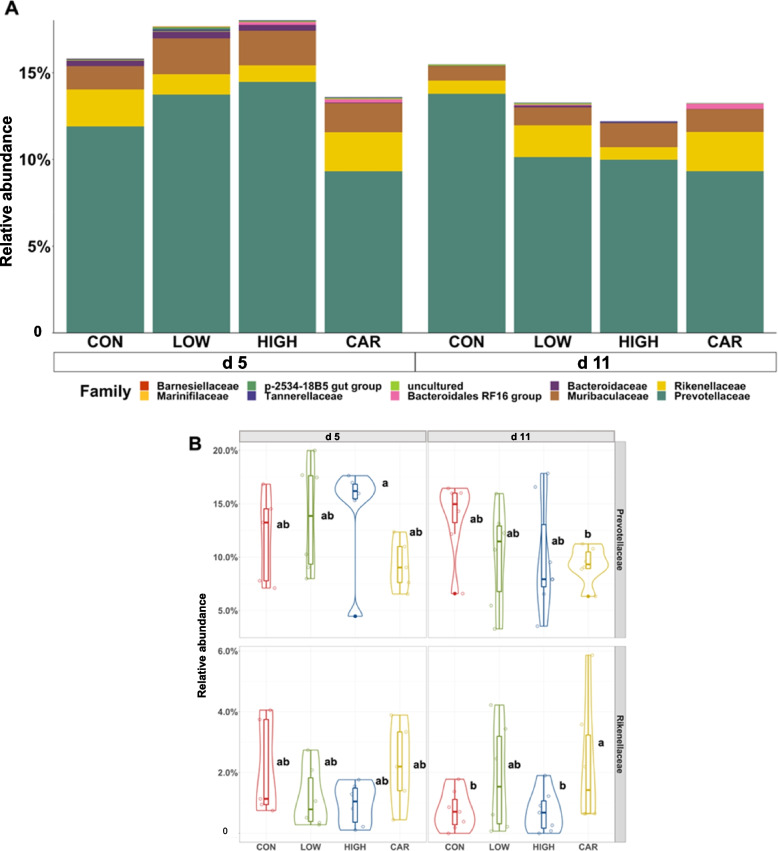
Stacked bar plot showing the relative abundance of Bacteroidetes family in colon digesta of enterotoxigenic *E. coli* F18 challenged pigs fed diets supplemented with different dose of oligosaccharide-based polymer active substance or carbadox on d 5 and 11 post-inoculation (**A**). Violin plot showing the relative abundance of individual bacterial phylum (**B**). ^a,b^Means without a common superscript are different (*P* < 0.05). Each least squares mean represents 6 observations. CON = Basal nursery diet (control); LOW = Control diet supplemented with 10 mg/kg of oligosaccharide-based polymer; HIGH = Control diet supplemented with 20 mg/kg of oligosaccharide-based polymer; CAR = Control diet supplemented with 50 mg/kg of carbadox

A total of 726,005 qualified reads were obtained with a mean of 15,122 reads per sample. No differences were observed in the alpha diversity of distal colon microbiota among dietary treatments on d 5 and d 11 PI (Fig. S3). Beta diversity (Adonis analysis based on the Bray–Curtis distance) indicated the interactive effect of treatment (diet) and day (d 5 PI vs. d 11 PI) was observed in the overall bacterial composition (Adonis, *P* < 0.05). Compositional differences of the distal colon microbiota were also observed between CON or LOW or HIGH and CAR on d 5 and d 11 PI (Pairwise-Adonis, *P* < 0.05; Fig. 4). The dominant phyla in distal colon digesta were Firmicutes, Bacteroidetes, Proteobacteria, and Actinobacteria, regardless of treatment or sampling day (Fig. S4). Pigs in the HIGH or CAR group had a lower (*P* < 0.05) relative abundance of Actinobacteria than pigs in the CON group on d 11 PI. Supplementation of HIGH reduced the relative abundance (*P* < 0.05) of Bacteroidetes in the distal colon on d 11 PI than on d 5 PI. Within the Firmicutes phylum (Fig. 5), pigs in the CAR group had the highest (*P* < 0.05) relative abundance of Clostridiaceae (17.14%) in the distal colon on d 11 PI, but the lowest (*P* < 0.05) relative abundance of Streptococcaceae (0.21%) or Lactobacillaceae (5.82%) on d 5 or d 11 PI among treatment groups, respectively. Pigs fed CAR had reduced the relative abundance (*P* < 0.05) of Lachnospiraceae (27.40% vs. 20.30%) in the distal colon on d 11 PI than d 5 PI. Within the Bacteroidetes phylum (Fig. 6), pigs in the CON and HIGH had reduced (*P* < 0.05) relative abundance of Rikenellaceae (0.76% and 0.72% vs. 2.27%) in distal colon on d 11 PI, compared with pigs in the CAR group.

## Discussion

ETEC infection can remarkably change metabolome [28, 29] and intestinal microbial profiles in pigs [30, 31]. However, there is still limited research on the impacts of different feed additives on various aspects of gut health and development in pigs, especially under disease-challenged conditions. The present study investigated the alteration of metabolic pathways in the serum and colon digesta by using an untargeted metabolomics approach when pigs were supplemented with oligosaccharide-based polymer or therapeutic levels of carbadox. Results from the current study demonstrated that major alteration of metabolic pathways in the serum and colon digesta were observed between the oligosaccharide-based polymer and antibiotic groups. Additionally, supplementation of antibiotic resulted in distinct modifications to microbial composition and community diversity in colon digesta of weaned pigs challenged with ETEC F18.

### Changes in metabolites in serum and distal colon

Recent advances in the high-throughput metabolomics analysis provide comprehensive approaches for identifying the impacts of specific bioactive compounds or disease on microbiota metabolites and host metabolic pathways [32]. In the current study, the metabolic profiles of serum and colon digesta were notably different in pigs supplemented with oligosaccharide-based polymer compared with antibiotic. These findings suggest that oligosaccharide-based polymer exhibited distinct effects on metabolic pathways in ETEC-infected pigs when compared with carbadox.

The use of in-feed antibiotics has shown promising results for performance enhancement in weaned pigs [33], partially due to increased nutrient digestibility, utilization, and metabolism [34]. In the present study, in-feed antibiotic carbadox primarily influenced serum metabolites that relate to amino acid metabolism, compared to pigs in the control diet (Fig. S2). This observation is consistent with our previous research [35] and research published by other labs [36, 37]. These alterations suggest that antibiotic treatment may increase amino acid availability for the host. For example, one of the most affected metabolic pathways was aminoacyl-tRNA biosynthesis, which is essential for charging amino acids to their cognate transfer RNAs (tRNA) to provide the substrates for protein synthesis [38]. The disruption of aminoacyl-tRNA biosynthesis including transcription regulation, signal transduction, angiogenesis, and other aspects of cellular homeostasis by antibiotic treatment could potentially result in altered protein synthesis rates and host cellular functions [39, 40]. Enhanced growth rate and disease resistance by antibiotic treatment in our previously published research [14] may be highly correlated with amino acid metabolic pathway modifications in supporting animal growth, maintaining homeostasis, and regulating other biological processes in the host [41, 42]. Further studies are warranted to understand the exact mechanisms underlying these alterations and their consequences for host physiology.

Interestingly, supplementation of oligosaccharide-based polymer had limited impacts on serum metabolites compared to the control, but significantly affected the metabolite profile in colon digesta of weaned pigs compared with carbadox. Specifically, addition of 20 mg/kg of oligosaccharide-based polymer increased the levels of indole-3-acetate (IAA) in distal colon digesta more than 7-fold during the peak of ETEC infection. IAA is one of the derivatives of tryptophan metabolism and is known to promote IL-22 production in the intestine [43]. IL-22 is known to act exclusively on intestinal epithelial cells, promoting cell regeneration and tissue repair [44]. In particular, IL-22 in the gastrointestinal tract stimulates epithelial cells to produce antimicrobial peptides, which enhances the intestinal barrier function against pathogens [45]. The protective role of IL-22 in the intestine of the pigs against ETEC was also supported by previously published studies [46, 47]. Furthermore, IAA has been reported to reduce the levels of TNF-α and IL-1β in the macrophages of mice when stimulated with lipopolysaccharide [48]. The observed increase in IAA abundance in colon digesta of pigs supplemented with oligosaccharide-based polymer may be attributed to the down-regulated mRNA expression of *TNFA*, *IL1B*, and *IL6* in ileal mucosa, as previously reported [14]. IAA can also be produced by various bacterial species, such as *Lactobacillus*, *Bacteroides*, and *Clostridium*, to inhibit the growth of potentially pathogenic bacteria, including *Salmonella* [49], *Shigella* [50], and *Escherichia* [51]. Therefore, the observed increase of IAA in the colon of pigs supplemented with oligosaccharide-based polymer might contribute to the enhancement of intestinal immune responses and modification of gut microbiota during the ETEC infection.

The secretory diarrhea is ascribed to the virulence factors of ETEC, such as heat-labile toxins, heat-stable toxins, Shiga toxins, and lipopolysaccharides [52]. Among these virulence factors, Shiga or Shiga-like toxin induces edema disease and hemorrhagic colitis, which can leads to gastrointestinal bleeding or the death of weaned pigs [53]. Tranexamic acid is a derivative of lysine used for reducing gastrointestinal hemorrhage in clinical practices [54]. The major protective effect of tranexamic acid is to inhibit fibrinolysis by blocking the interaction of plasminogen with the lysine residues of fibrin [55]. Moreover, tranexamic acid was reported to be involved in various biological processes, including protein synthesis, metabolic signaling activities via acetylation, protein–protein interactions, and post-translational modifications in immune and inflammatory responses [56]. In the present study, pigs supplemented with oligosaccharide-based polymer contained 12-fold more tranexamic acid in distal colon digesta compared with carbadox pigs during the peak of ETEC infection. The escalated tranexamic acid in pigs supplemented with oligosaccharide-based polymer may contribute to the enhanced disease resistance and performance of ETEC-challenged pigs.

Significant changes in amino acid metabolism were also observed in the distal colon digesta when comparing pigs supplemented with oligosaccharide-based polymer to those given carbadox. These changes include arginine biosynthesis, arginine and proline metabolism, and glutamine and glutamate metabolism. Arginine and proline play important roles in wound healing, antioxidative reactions, immune responses, and protein biosynthesis [57, 58]. Arginine is crucial for the host defense system, as its metabolite, nitric oxide, has demonstrated antimicrobial activity against pathogens [57]. Arginine-derived polyamines and pyrroline-5-carboxylate from proline metabolism are also critically important in regulating cellular signaling pathways and stimulating gut mucosal immune cells’ maturation [59, 60]. Additionally, glutamine and glutamate are essential energy source for porcine intestinal mucosal cells and are intricately connected to both arginine and proline metabolism as specific precursors [61, 62]. These findings suggest that the enhancement of gut immunity by supplementing oligosaccharide-based polymer might also be attributed to the changes in those specific amino acid metabolism [14].

### Changes in microbial community in distal colon

The composition of the microbial community in the pig's intestine can be influenced and shifted by several factors, including diet, age, health status, and environmental conditions [63, 64]. Research also reported that the intestinal microbial diversity and composition was affected by in-feed antibiotics [63, 65] and other feed additives [66, 67]. In consistent with previously published research [17, 63], the present study confirmed that carbadox supplementation significantly altered the colonic microbiota composition and diversity compared with control. However, there were no clear separation nor significant changes in the relative abundance of microbial compositions between pigs supplemented with oligosaccharide-based polymer vs. control.

During the peak of ETEC infection period (d 5 PI), the relative abundance of Streptococcaceae within the Firmicutes phylum was reduced in colon digesta of pigs fed antibiotic compared to those supplemented with oligosaccharide-based polymer and control. Streptococcaceae are Gram-positive bacteria, and some species can be opportunistic pathogens that cause diseases in pigs [68]. For instance, *Streptococcus suis* (*S. suis*) is classified within the family of Streptococcaceae, which is associated with a wide range of diseases in pigs, such as meningitis, septicaemia, pneumonia, endocarditis, and arthritis [69]. It has been reported that weanling stress increased the abundance of potentially harmful *S. suis* in intestinal contents [70]. Additionally, *S. suis* is capable of invading multiple organs to cause exaggerated inflammation, which has to be treated with antibiotics [71]. The reduced abundance of Streptococcaceae in colon digesta of pigs was also observed in our previous experiment when carbadox was administered [35].

During the recovery period (d 11 PI), the relative abundance of Clostridiaceae and Rikenellaceae were increased, whereas Lactobacillaceae was reduced in colon digesta of pigs fed with carbadox, compared with pigs in control and pigs supplemented with oligosaccharide-based polymer. Clostridiaceae is known to correlate with the production of short-chain fatty acids [72] and improved weight gain in pigs [73]. Rikenellaceae has been reported to facilitate the breakdown of proteins and carbohydrates in feed and been more abundant in high feed conversion ratio pigs [74]. The increased Clostridiaceae and Rikenellaceae in colon support the growth promoting effects of carbadox [35]. However, supplementing carbadox reduced the relative abundance of Lactobacillaceae in colon digesta compared with other treatments. Lactobacillaceae is usually considered as one of favorable bacteria in the intestine to maintain gut microbiota balance by inhibiting the growth of pathogens [75, 76]. The relative abundance of *Lactobacillus* is generally enriched after weaning, as these bacteria have the ability to break down complex plant-based carbohydrates into simpler sugars for energy production [77, 78]. In agreement with other published research, the reduced Lactobacillaceae and other beneficial bacteria due to antibiotic administration might increase the risk of intestinal dysbiosis and overall health [79, 80]. Although oligosaccharide-based polymer and carbadox exhibited similar growth-promoting benefits in the present study [14], their impacts on the distal colon microbiota were different. The changes in gut bacterial biomass are unknown due to the limits of 16S rRNA sequencing. In future research, it is advisable to investigate the spatial impacts, particularly the changes in the microbiota composition in the ileum where ETEC colonizes.

## Conclusions

The present study identified differential metabolites and their pathways in the serum and distal colon digesta of ETEC F18 challenged pigs, fed either an oligosaccharide-based polymer or an antibiotic. In comparison to the antibiotic, the oligosaccharide-based polymer had an impact on several important metabolomic markers associated with immune responses and nutrient metabolism in the distal colon, including IAA and tranexamic acid. The impacted metabolic pathways, including those involving arginine, glutamine and glutamate metabolism, may be associated with enhanced disease resistance and intestinal immune responses in weaned pigs. Interestingly, when compared with carbadox supplementation, the impacts of the oligosaccharide-based polymer on microbiota composition in distal colon of weaned pigs were limited. To better understand the mechanisms by which this polymer controls post-weaning diarrhea, further research is recommended to focus on the small intestine and the interactions of host, local immunity, and microbiota.

### Supplementary Information

**Additional file 1: Fig. S1.** Partial least squares discriminant analysis (PLS-DA) 2D score plot of the metabolites in serum showed separated clusters between the CON and CAR groups on d 5 (**A**) and d 11 post-inoculation (**B**). Partial least squares discriminant analysis (PLS-DA) 2D score plot of the metabolites in distal colon digesta showed separated clusters between the CON and 10 or 20 mg/kg oligosaccharide-based polymer active substance groups on d 11 post-inoculation (**C**). CON = Basal nursery diet (control). LOW = Control diet supplemented with 10 mg/kg of oligosaccharide-based polymer. HIGH = Control diet supplemented with 20 mg/kg of oligosaccharide-based polymer. CAR = Control diet supplemented with 50 mg/kg carbadox. Shaded areas in different colors represent in 95% confidence interval. **Fig. S2.** Significantly changed pathways (−log_10_(*P*) > 1.5) in serum between the control and 50 mg/kg carbadox on d 5 (**A**) or d 11 post-inoculation (**C**). The *x*-axis represents the pathway impact values and the *y*-axis represents the −log(*P*) values from the pathway enrichment analysis. Metabolite set enrichment analysis (**B** and **D**) shows the metabolic pathways were altered in control compared to 50 mg/kg carbadox on d 5 or d 11 post-inoculation, respectively. Both pathway analysis and metabolite set enrichment analysis were performed using identified metabolites with VIP > 1. **Fig. S3.** Alpha diversity as indicated by Shannon (**A**) and Chao 1 (**B**) in distal colon digesta of enterotoxigenic *E. coli* F18 challenged pigs fed diets supplemented with different dose of oligosaccharide-based polymer active substance or antibiotics (carbadox) on d 5 and 11 post-inoculation. ^a–c^Means without a common superscript are different (*P* < 0.05). Each least squares mean represents 6 observations. CON = Basal nursery diet (control); LOW = Control diet supplemented with 10 mg/kg oligosaccharide-based polymer active substance; HIGH = Control diet supplemented with 20 mg/kg oligosaccharide-based polymer active substance; CAR = Control diet supplemented with 50 mg/kg carbadox. **Fig. S4.** Stacked bar plot showing the relative abundance of bacterial phyla in colon digesta of enterotoxigenic *E. coli* F18 challenged pigs fed diets supplemented with different dose of oligosaccharide-based polymer active substance or antibiotics (carbadox) on d 5 and 11 post-inoculation (**A**). Violin plot showing the relative abundance of individual bacterial phylum (**B**). ^a,b^Means without a common superscript are different (*P* < 0.05). Each least squares mean represents 6 observations. CON = Basal nursery diet (control); LOW = Control diet supplemented with 10 mg/kg oligosaccharide-based polymer active substance; HIGH = Control diet supplemented with 20 mg/kg oligosaccharide-based polymer active substance; CAR = Control diet supplemented with 50 mg/kg carbadox.

## Data Availability

All data generated or analyzed during this study are available from the corresponding author upon reasonable request.

## Abbreviations

ETEC: Enterotoxigenic *E. coli*
FDR: False discovery rate
MIC: Minimal inhibitory concentrations
OTU: Operational taxonomic units
PCoA: Principal coordinates analysis
PI: Post-inoculation
PLS-DA: Partial least squares discriminant analysis
VIP: Variable importance in the projection
