# Supplementary material for: Impact of an oligosaccharide-based polymer on the metabolic profiles and microbial ecology of weanling pigs experimentally infected with a pathogenic E. coli
Source: J Anim Sci Biotechnol. 2024 Jan 2;15:1. doi: 10.1186/s40104-023-00956-8 (PMC10759389; doi:10.1186/s40104-023-00956-8)
Supplement: Supplementary file 1 — Additional file 1: Fig. S1. Partial least squares discriminant analysis (PLS-DA) 2D score plot of the metabolites in serum showed separated clusters between the CON and CAR groups on d 5 (A) and d 11 post-inoculation (B). Partial least squares discriminant analysis (PLS-DA) 2D score plot of the metabolites in distal colon digesta showed separated clusters between the CON and 10 or 20 mg/kg oligosaccharide-based polymer active substance groups on d 11 post-inoculation (C). CON = Basal nursery diet (control). LOW = Control diet supplemented with 10 mg/kg of oligosaccharide-based polymer. HIGH = Control diet supplemented with 20 mg/kg of oligosaccharide-based polymer. CAR = Control diet supplemented with 50 mg/kg carbadox. Shaded areas in different colors represent in 95% confidence interval. Fig. S2. Significantly changed pathways (−log10(P) > 1.5) in serum between the control and 50 mg/kg carbadox on d 5 (A) or d 11 post-inoculation (C). The x-axis represents the pathway impact values and the y-axis represents the −log(P) values from the pathway enrichment analysis. Metabolite set enrichment analysis (B and D) shows the metabolic pathways were altered in control compared to 50 mg/kg carbadox on d 5 or d 11 post-inoculation, respectively. Both pathway analysis and metabolite set enrichment analysis were performed using identified metabolites with VIP > 1. Fig. S3. Alpha diversity as indicated by Shannon (A) and Chao 1 (B) in distal colon digesta of enterotoxigenic E. coli F18 challenged pigs fed diets supplemented with different dose of oligosaccharide-based polymer active substance or antibiotics (carbadox) on d 5 and 11 post-inoculation. a–cMeans without a common superscript are different (P < 0.05). Each least squares mean represents 6 observations. CON = Basal nursery diet (control); LOW = Control diet supplemented with 10 mg/kg oligosaccharide-based polymer active substance; HIGH = Control diet supplemented with 20 mg/kg oligosaccharide-based polymer active subst [file 40104_2023_956_MOESM1_ESM.pdf]

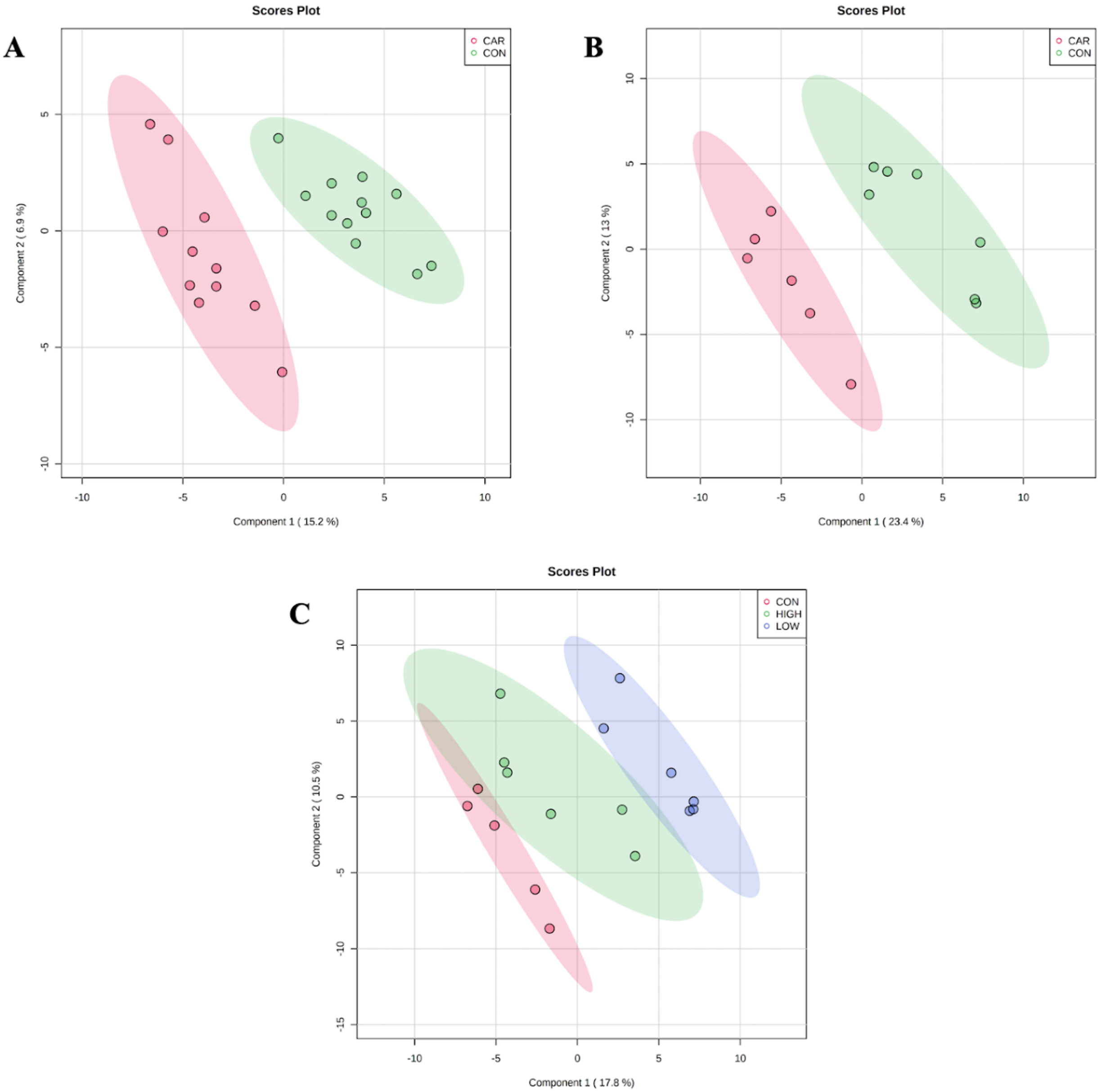

Fig. S1 Partial Least Squares Discriminant Analysis (PLS-DA) 2D score plot of the metabolites in serum showed separated clusters between the CON and CAR groups on d 5 (**A**) and d 11 (**B**) post-inoculation. Partial Least Squares Discriminant Analysis (PLS-DA) 2D score plot of the metabolites in distal colon digesta showed separated clusters between the CON and 10 or 20 mg/kg oligosaccharide-based polymer active substance groups on d 11 post-inoculation (**C**). CON = Basal nursery diet (control). LOW = Control diet supplemented with 10 mg/kg of oligosaccharide-based polymer. HIGH = Control diet supplemented with 20 mg/kg of oligosaccharide-based polymer. CAR = Control diet supplemented with 50 mg/kg carbadox. Shaded areas in different colors represent in 95% confidence interval

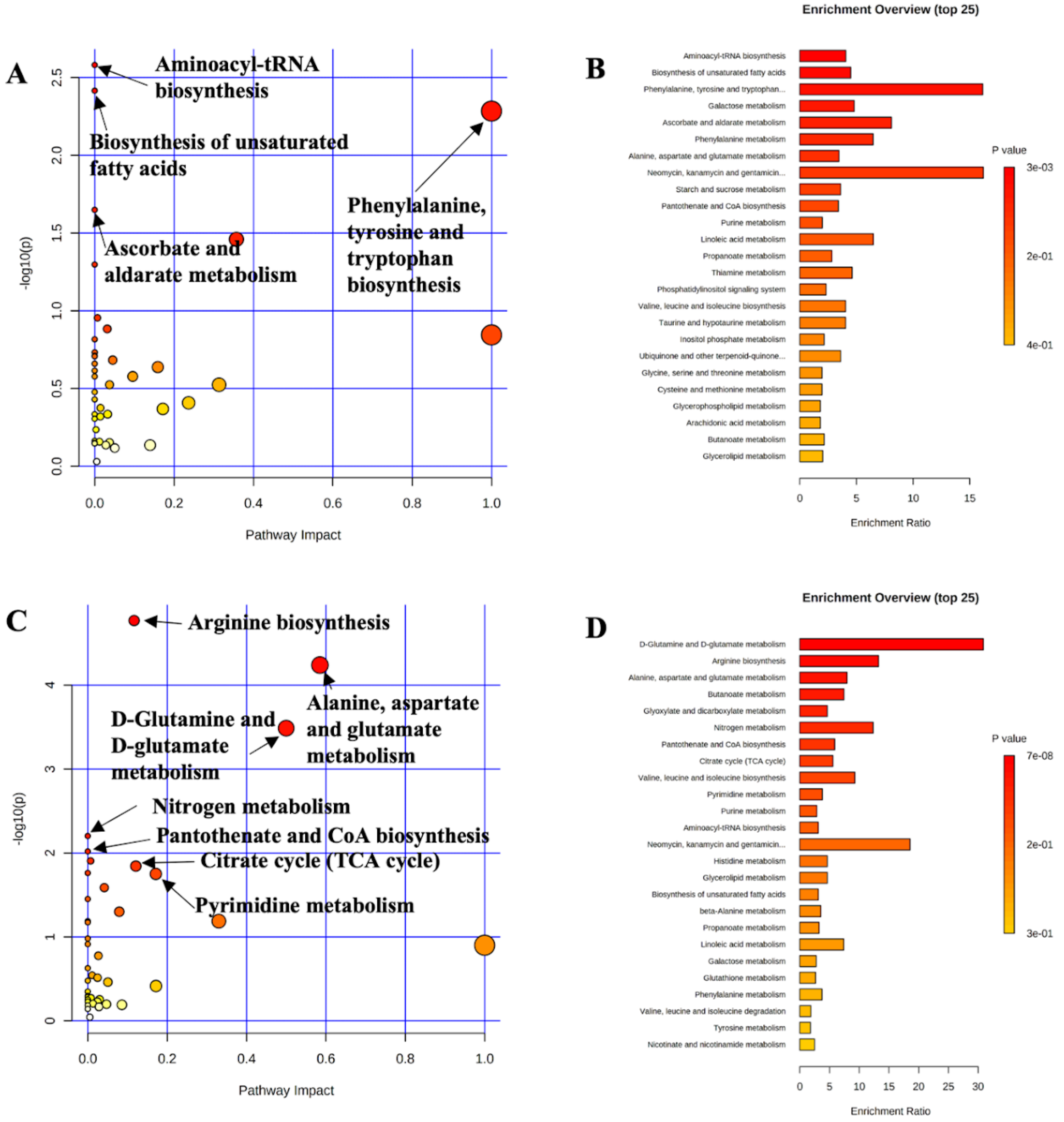

Fig. S2 Significantly changed pathways (−log *P* value > 1.5) in serum between the control and 50 mg/kg carbadox on d 5 (**A**) or d 11 (**C**) post-inoculation. The *x*-axis represents the pathway impact values and the *y*-axis represents the −log(*P*) values from the pathway enrichment analysis. Metabolite set enrichment analysis (**B** and **D**) shows the metabolic pathways were altered in control compared to 50 mg/kg carbadox on d 5 or d 11 post-inoculation, respectively. Both pathway analysis and metabolite set enrichment analysis were performed using identified metabolites with VIP > 1

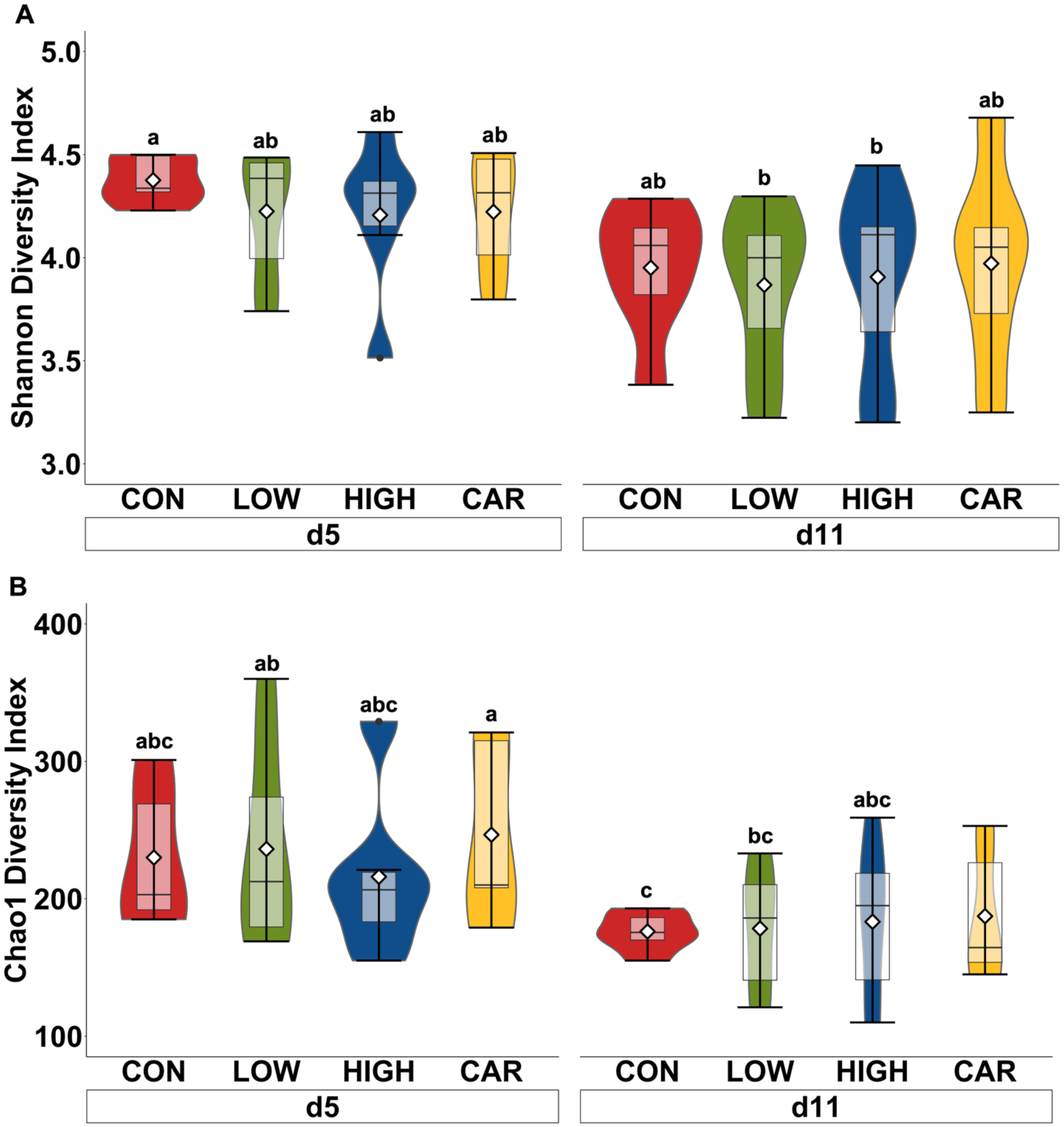

Fig. S3 Alpha diversity as indicated by Shannon (**A**) and Chao 1 (**B**) in distal colon digesta of enterotoxigenic *E. coli* F18 challenged pigs fed diets supplemented with different dose of oligosaccharide-based polymer active substance or antibiotics (carbadox) on d 5 and 11 post-inoculation. [a-c]Means without a common superscript are different ($P < 0.05$). Each least squares mean represents 6 observations. CON = Basal nursery diet (control); LOW = Control diet supplemented with 10 mg/kg oligosaccharide-based polymer active substance; HIGH = Control diet supplemented with 20 mg/kg

oligosaccharide-based polymer active substance; CAR = Control diet supplemented with 50 mg/kg carbadox

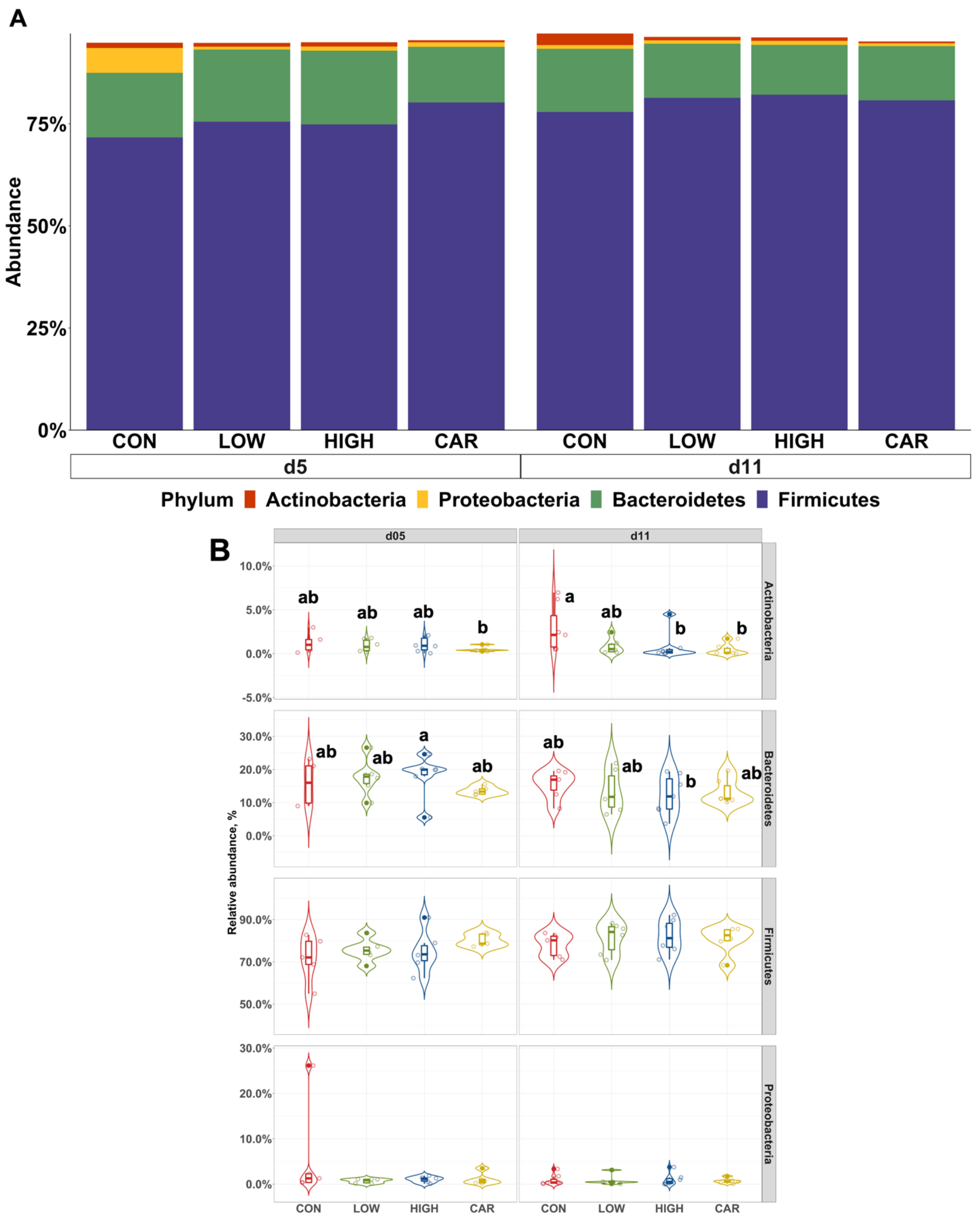

Fig. S4 Stacked bar plot showing the relative abundance of bacterial phyla in colon digesta of enterotoxigenic *E. coli* F18 challenged pigs fed diets supplemented with different dose of oligosaccharide-based polymer active substance or antibiotics (carbadox) on d 5 and 11 post-inoculation

(A). Violin plot showing the relative abundance of individual bacterial phylum **(B)**. [a,b]Means without a common superscript are different ($P < 0.05$). Each least squares mean represents 6 observations. CON = Basal nursery diet (control); LOW = Control diet supplemented with 10 mg/kg oligosaccharide-based polymer active substance; HIGH = Control diet supplemented with 20 mg/kg oligosaccharide-based polymer active substance; CAR = Control diet supplemented with 50 mg/kg carbadox
